# Legitimate or Not, Does It Really Matter? A Reading of the PDO Label’s Legitimacy through Consumers’ Perception

**DOI:** 10.3390/foods12122365

**Published:** 2023-06-14

**Authors:** Maria Bouhaddane, Rafia Halawany-Darson, Corinne Rochette, Corinne Amblard

**Affiliations:** 1 CIRAD, UMR Innovation, Univ. Montpellier, CIRAD, INRAE, Institut Agro, F-34398 Montpellier, France; 2 Université Clermont Auvergne, INRAE, VetAgro Sup, UMR 545 Fromage, 63370 Lempdes, France; rafia.halawany-darson@vetagro-sup.fr (R.H.-D.); corinne.amblard@vetagro-sup.fr (C.A.); 3 Clermont Research Management Center, Health and Territory Research Chair of University of Clermont Au-vergne, 63000 Clermont-Ferrand, France; corinne.rochette@uca.fr

**Keywords:** legitimacy, perceived quality, purchase intention, food consumption behavior, PDO label, Partial Least Square Structural Equation Modeling (PLS-SEM), labeling schemes

## Abstract

The proliferation of quality labels for the same food product questions the relevance of labeling schemes. Based on the theory of legitimacy and research on food-related consumer behavior, this study aims to examine the influence of the perceived legitimacy of a label (PDO) on consumers’ perceptions of the quality and purchase intentions of the labeled product. A conceptual model was, therefore, developed to estimate the influence of four dimensions of legitimacy on the perceived quality and purchase intention of PDO-labeled cheese, French cheeses being products whose quality is traditionally linked to their regional origin. Our model was tested on a sample of 600 consumers representative of the French population. Using Partial Least Square Structural Equation Modeling, results show that for surveyed consumers, the pragmatic, regulative, and moral legitimacy of the PDO label positively influences the perceived quality of PDO-labeled cheese. Furthermore, pragmatic legitimacy has a substantial and direct influence on purchase intention, whereas both regulative and moral legitimacy influence purchase intention only indirectly through perceived quality. Unexpectedly, our findings do not show a significant influence of cognitive legitimacy either on perceived quality or purchase intention. The output of this research contributes to a better understanding of the link between a label’s legitimacy, perceived quality, and purchase intention.

## 1. Introduction

The increasing demand for high-quality food products, as well as the growing concern for health, environment, and social responsibility, make labeling an important tool for differentiation. Quality labels represent an opportunity for food companies to signal the presence of desirable attributes to buyers [1] and to reinforce their positioning in the market [2,3].

The informational role of labels is widely acknowledged in the literature [4,5,6]. In general, they can help to transform complex information about product characteristics into simple logos in order to simplify consumers’ decision process [7]. From a signaling theory perspective [8], labels are a means of converting experience attributes (attributes that can only be evaluated by consumers after the product has been used or consumed) or credence attributes (characteristics that consumers are not in a position to verify) into search attributes (characteristics that are easily observable by consumers) [9,10]. They can, therefore, act as an informational shortcut in consumers’ information processing activity [11].

By revealing the unobservable product characteristics to the consumers, labels would reduce their uncertainties about the quality of the offer [1,12,13,14]. However, labels are much more complex devices that represent a shared framework for interpreting and evaluating the qualities of a product. The set of beliefs (descriptive and inferential) generated by a label could mediate the effect of the latter on the perceived quality of the labeled product [15] and on the judgment made by consumers.

By combining the contributions of information economics and the signaling theory [16,17,18,19] on the one hand, and those of the convention theory [20,21,22,23,24] on the other, labels can be defined as signals of a quality constructed on the basis of quality conventions, which result from a collective cognitive process of qualification. These conventions linking producers and consumers are formalized in a set of specifications that the label guarantees by means of third-party certification.

Many empirical studies have assessed the effects of labels on the perceived quality of labeled products [12,15,25,26]. This literature shows that, in general, labels are perceived as relevant quality signals and that their presence informs of a higher quality. However, in recent years, there has been a proliferation of labels on the same product. This practice has grown, and it is no longer unusual to see up to three labels on a food product [7,27,28]. Their perception by consumers can, therefore, be questioned [29]. Indeed, if labels carry different messages and promises, either they can be perceived as complementary and self-reinforcing, or they can lead to a blurring and dilution of the messages conveyed to consumers. The existence of multiple labels raises the question of whether there is a perceived hierarchy between the value of these labels and of the legitimacy granted to them by consumers.

Moreover, since it is difficult for consumers to assess the validity of the promises made about the quality of the labeled product (i.e., unobservable attributes) [30], the initial uncertainty about the quality of the product is then projected onto the credibility of the labeling scheme [31] and the third-party certifiers. The intervention of a third party, external to the market exchange, in the labeling process makes it possible to legitimize the qualities stated by the label and for producers to use the label to maintain a certain power over the market. Hence, the perceived legitimacy of the label probably depends on the perceived legitimacy of the certifying body (third-party certification) that delivers the authorization or t agreement to use the label on a product [32]. In food labeling research, the challenge of perceived legitimacy is an important issue [33] that has received little attention from scholars.

In order to fill this gap in the literature, we propose an original reading of the perceived quality and purchase intention of labeled food products based on four types of legitimacy. Our research aims to answer the following question: To what extent does the perceived legitimacy of a label (PDO), from the consumer’s perspective, influence the perceived quality of the labeled food product and the intention to purchase it? To answer this question, cheese was selected as the product for the study of the PDO label as it represents a category of food products for which quality is traditionally linked to the region of origin (terroir), especially in France, which has a long-standing tradition of producing and labeling regional cheeses (with 46 PDOs). This paper is organized as follows: the next section presents the contributions of research on food labeling in relation to perceived quality and purchase intention and reviews the existing literature on legitimacy. Then, our research hypotheses, followed by the conceptual model and the methodology, are developed. The main results of our study are subsequently presented and discussed. Finally, our paper concludes with some practical implications for producers and public institutions.

## 2. Literature Review and Conceptual Model

### 2.1. Influence of Quality Labels on Perceived Quality and Purchase Intention

With consumers’ and public authorities’ increasing interest in food quality, a number of international food standards and voluntary quality labeling schemes emerged in the current globalized food supply chain. Quality labels are a way for companies to signal the presence of unobservable desirable attributes to buyers [1] and to strengthen their positioning and the differentiation of their products on the market [2]. They are a tool to communicate and reduce the information asymmetry between producers and consumers. Quality labels are the result of a collective process of construction and concretization of a project driven by public or private actors in search of differentiation [34]. Moreover, they lay down a set of quality specifications and a process for assessing compliance with the requirements, which they guarantee to the public. Hence, quality labels have a dual mission of providing information and guaranteeing compliance with some production and quality criteria to the consumer. Furthermore, these labels have the potential to ensure social and environmental sustainability in the food supply. Indeed, over the last several decades, voluntary quality schemes have become a key approach to promoting sustainable value chains for agricultural commodities, and they play a significant role in helping food businesses meet the 2030 Sustainable Development Goals (SDGs).

In addition to the EU quality schemes (PDO/PGI/TSG—Regulation (EU) No 1151/2012), a large number of private and national food labels or quality schemes exist, covering a wide range of initiatives in terms of their scope, objectives, and operational methods. An important distinction between these quality schemes is whether or not they rely on a third-party certification procedure, thus grouping them into self-declaration schemes (which operate on the market on the basis of a label or a logo, often registered as a trademark) on the one hand, and certification schemes, on the other.

Nowadays, consumers purchase foods from certified sources in order to access high-quality products [35]. Indeed, certification schemes for agricultural and food products provide assurance (through a certification mechanism) that certain attributes of the product or its production method, as defined in a book of specifications, have been complied with. They are complex procedures that involve many actors and stakeholders. A certification mechanism requires a conformity assessment body that plays the role of the certifier. The latter verifies (or appoints an external assessor to ensure) that the object of evaluation complies with the certification requirements and decides whether or not to grant the certification (written assurance). Despite the fact that there is no standardized certification framework, certification in many agri-food supply chains has become an important means to reinforce compliance with quality and process acceptability standards, including labor rights, social conditions of agricultural production, environmental aspects, and food security [36].

Within this framework, the PDO label acts as a guarantee that the product has been produced based on a specific know-how and processed in a defined geographical area, using recognized techniques and particular specifications inspected by public authorities and independent third-party bodies. It belongs to the category of labels with a tripartite guarantee system, involving a prescriber who is independent of the producer, and of the controller (who is accredited by an accreditation body).

Previous studies showed that quality labels increase consumers’ preferences and willingness to pay for a food product because they are perceived as quality signals [14,28,37]. Hence, the economic signaling theory emphasizes the importance of the credibility of the signal [38] and the confidence placed in it by consumers [39]. Indeed, the use of information does not only depend on its mere content but also on the level of confidence in the source of information [40]. Consumers have more trust in food products labeled with the Protected Designation of Origin (PDO) or Geographical Indication (GI). This probably results from the greater perceived control by food authorities for this type of food products [41]. Higher confidence in the EU PDO labeling system is significantly positively correlated with the intention to purchase the PDO cheese [42]. Purchase intention indicates the probability of future purchases declared by a consumer. Bagozzi and Burnkrant [43] distinguish purchase intention from purchase desire by the fact that it is consumers’ subjective tendency to pay for the products or services. It is a conative/behavioral aspect that cannot be separated from the process of purchasing decisions [44]. This intention of making a purchase is influenced by the understanding of the product [45] and is also the expression of a combination of many external or internal factors, such as personal, social, economic, and psychographic variables. The performance and effectiveness of quality labels are often associated with their ability to influence consumer preferences and purchase intention [6]. The latter can predict future consumer behavior or purchase [46].

However, in the context of the multiplication of labels on food products and food fraud scandals, and with the media reporting certain failings or opportunistic behavior on the part of suppliers and manufacturers, the question of labels’ perceived legitimacy and its influence on the perceived quality and purchase intention is worth examining.

### 2.2. Theoretical Approaches of Legitimacy

In a world where all actors, organizations, and consumers alike are seeking to increase their power over the market and society, the issue of legitimacy, which has its roots in the work of Weber [47], is particularly acute [48,49,50,51,52]. Legitimacy is inseparable from power and authority. An organization has power if its actions influence society and the behavior of individuals. The use of labels by organizations is a way of acquiring greater power in the market. Thus, quality labeling schemes are becoming an important global governance instrument. Several authors have discussed the legitimacy of these systems [53]. Dendler [54] emphasizes the importance of legitimacy for the institutionalization of social entities aimed at establishing a new “social order” [47], with product-labeling systems being a prominent example of such entities in contemporary societies. This resonates with many other authors who have stressed the importance of the legitimacy of product-labeling systems [55,56], including food and organic labels [57].

For an individual, legitimacy is the fact of perceiving an action as desirable within a system of socially constructed norms, values, and beliefs [49], thus contributing to the common welfare [58]. Legitimacy is, therefore, a judgment. It is a subjective notion. It refers to the capacity of an organization to fulfill its promises through the actions it undertakes. Thus, legitimacy means that the necessary means to achieve the objectives of an organization, and these objectives themselves are in harmony with the goals and needs recognized as desirable in a given society. It is evolutionary because the legitimacy given by stakeholders depends on the organization’s ability to convince them that its actions are consistent with the values carried by the social system. Legitimacy can be considered from a strategic or institutional perspective. The strategic approach [59,60,61] views legitimacy as a manipulable resource [61] produced by the organization in order to increase its influence on the market. The label is used to gain a more prominent position in the market. From this point of view, the label constitutes a constructed strategic resource, a differentiating factor, carrying a promise or even a particular ideology (e.g., organic, ethical or halal labels). The institutional approach [62,63,64] depends on how external stakeholders, including consumers, view the object of the action and its conformity to a system of shared social values [65]. In this endeavor, the role of the certifier and the credit and trust granted to it is central. In this respect, legitimacy is a form of assent expressed by consumers toward the organization. The company’s action is perceived as being consistent with the societal principles that people are collectively attached to. The two approaches interact in order for the actions developed by the organizations to be considered legitimate. More specifically, organizations will attempt to activate different types of legitimacy in order to obtain collective approval for their actions: the socio-political legitimacy, which is itself divided into regulative, pragmatic, and normative dimensions, and the cognitive legitimacy (Table 1). Socio-political legitimacy refers to the fact that stakeholders accept an action, an approach, or a project because it conforms to the prevailing laws and standards [66]. It is based on a regulative dimension (conformity to norms and to the legal framework) but also a pragmatic dimension (evaluation by the stakeholders of the benefits provided) and a moral (or normative) dimension referring to a positively perceived way of proceeding. Cognitive legitimacy, on the other hand, reflects a tacit understanding and does not translate into an active evaluation by stakeholders. It refers to the familiarity, understandability, and self-evident nature of the action or process [49,67]. This cognitive approach refers to the passive behavior of people [68].

Organizations, therefore, seek to acquire this legitimacy in order to increase their influence on the market and society, but do we know how this legitimacy is built from the consumers’ point of view when it comes to quality labels?

### 2.3. Hypotheses

In light of the previous considerations, this paper advances our understanding of the perceived legitimacy of labels by examining its link with the perceived quality and purchase intention of the labeled food product. For this purpose, we propose to measure the perceived legitimacy [49,52,65,66,67,69] of the PDO label through its four dimensions (pragmatic, regulative, moral, and cognitive).

Perceptions of legitimacy have been found to influence consumer decisions insofar as a higher level of perceived legitimacy increases positive evaluations of a firm [49,59]. For example, previous research [67,71] shows that an organization’s perceived legitimacy has a positive effect on consumers’ attitudes and can bias their evaluations. Similarly, it can be expected that the perceived legitimacy of a label intervenes in the evaluation of the quality of the labeled product. A halo effect [72] could occur when a label is perceived as legitimate, leading the consumer to infer the desired quality of the labeled product. Hence, the following hypotheses are proposed:

**H1a.**  *The pragmatic legitimacy of the label positively influences the perceived quality of the labeled food product*;

**H1b.**  *The regulative legitimacy of the label positively influences the perceived quality of the labeled food product*;

**H1c.**  *The moral legitimacy of the label positively influences the perceived quality of the labeled food product*;

**H1d.**  *The cognitive legitimacy of the label positively influences the perceived quality of the labeled food product*.

Furthermore, it is widely acknowledged in the literature that legitimacy induces positive outcomes for organizations [73,74,75], which result in public support and positive behavioral intentions (purchase intention, positive word-of-mouth) [76]. The effect of legitimacy on purchase intention has been explained in light of the resource dependence theory [49,59] provided that consumers, given their limited resources, are likely to choose and purchase products from firms they consider legitimate [71,77]. By extending this to quality labels, we can assume that consumers would be more likely to buy a labeled product when the label displayed on the product is perceived as legitimate. Therefore, we postulate the following:

**H2a.**  *The pragmatic legitimacy of the label positively influences the purchase intention of the labeled food product*;

**H2b.**  *The regulative legitimacy of the label positively influences the purchase intention of the labeled food product*;

**H2c.**  *The moral legitimacy of the label positively influences the purchase intention of the labeled food product*;

**H2d.**  *The cognitive legitimacy of the label positively influences the purchase intention of the labeled food product*.

Finally, previous research has shown the positive role of perceived quality on purchase intention [78]. This is illustrated, for example, by the findings of Moussa and Touzani [26], which confirm the positive influence of consumers’ perceived quality on the intention to purchase a labeled product. Therefore, the following hypothesis can be presented:

**H3.**  *The perceived quality of the labeled food product positively influences its purchase intention*.

**Figure 1:**
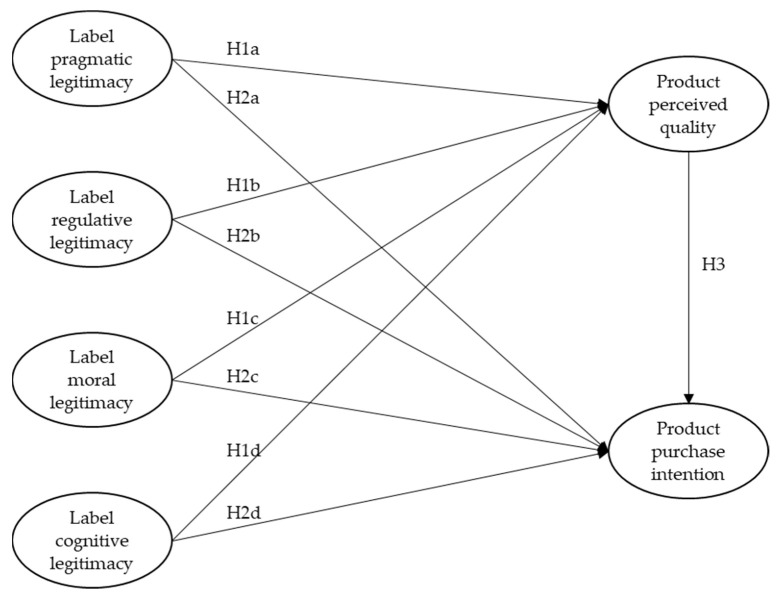
Conceptual model and hypotheses.

Based on these hypotheses, this research develops a conceptual model (Figure 1) to examine the influence of the different dimensions of a label’s perceived legitimacy (pragmatic, regulative, moral, and cognitive) on the perceived quality and purchase intention of the labeled product.

## 3. Materials and Methods

### 3.1. Measures and Questionnaire

In this research, perceived legitimacy was conceptualized as a second-order formative construct that integrates the four legitimacy dimensions as first-order constructs, which, in turn, consist of formative measurement [79]. Bouhaddane [70] developed the indicators for the measure of pragmatic, regulative, moral, and cognitive legitimacy. These indicators are reported in Table 2. Each item was measured on a 5-point Likert scale (1 = strongly disagree; 5 = strongly agree). As for the perceived quality and purchase intention of the labeled food product, they were each measured with three items adapted, respectively, from Larceneux [80] and Ingarao et al. [81]. These items are also presented in Table 2.

The questionnaire (cf. Appendix A) was written in French and consisted of 33 questions structured into three sections: (a) sociodemographic characteristics (seven questions); (b) consumers’ perceptions of PDO label’s legitimacy (twenty questions); (c) consumers’ perceptions of PDO cheese quality (three questions); and purchase intention (three questions). Additionally, three screening questions were included at the beginning of the survey to ensure that respondents were both buyers and consumers of cheese and that they were familiar with the PDO label.

A face-to-face pretest of our questionnaire was conducted with 10 cheese consumers to ensure that the instructions were understood and to avoid ambiguity in the questions. Minor adjustments, mainly in terms of wording, were made after this pre-test.

### 3.2. Data Collection and Sampling

The survey was conducted online from 25 February to 4 March 2021 with a panel of 600 French consumers. Data collection was executed by Toluna, a provider of survey panelists, which allowed for easier and quicker access to a large, heterogeneous sample of consumers. Participation in the survey was voluntary, and all data were anonymized. To ensure a representative sample of the French population, proportional quota sampling was used. Quotas were set for age, gender, region, and socio-professional category (based on the available census data) so as to recruit respondents in the same proportions as in the French population. Being a non-probability method, quota sampling can present potential biases which limit the generalizability of results. Nevertheless, as argued by Marsh and Scarbrough [82], no significant differences that would indicate considerable data biases were found when comparing quota sampling to random sampling.

### 3.3. Data Analysis

In order to assess the measurement model and to test our hypotheses, Partial Least Squares Structural Equation Modeling (PLS-SEM) was performed using SmartPLS 4 software. PLS-SEM was deemed suitable given the exploratory nature of our research model, which investigates conceptual relationships that have not yet been explored in the literature on food labels. Moreover, PLS-SEM is recommended for the estimation of models which include both reflective and formative constructs [83,84,85]. In reflective models, the observed variables (measurement indicators) are assumed to be caused by the latent variable (cf. Churchill, 1979). Indicators are, therefore, the manifestations that reflect the underlying construct. Thus, any variation in the value of the latent variable leads to a variation in the values of all its reflective indicators [86]. As a consequence, the relationship between the construct and its indicators is represented as follows:

$$x_{i}=\lambda_{i}\eta +\varepsilon_{i}$$

where *x_i_* is the *i*th indicator of the latent variable *η*; *ε_i_* is the measurement error for the *i*th indicator, and *λ_i_* is the coefficient representing the effect of *η* on *x_i_*.

In contrast, formative measurement models assume the opposite direction of the causal relationship between the latent variable and its manifest indicators, such that the indicators form or cause the construct [87], which implies the following relationship:

$$\eta =\overset{n}{\underset{i=1}{\sum }}\gamma_{i}x_{i}+\zeta$$

where *γ_i_* is the coefficient capturing the effect of indicator *x_i_* on the latent variable *η*, and *ζ* is a disturbance term.

The significance of the path coefficients in the structural model of PLS-SEM was assessed using the 5000 subsamples bootstrapping method [88].

## 4. Results

### 4.1. Sample Description

The detailed characteristics of our sample are presented in Table 3. In terms of participants’ gender, 52% were female, while 48% were male. Respondents’ age ranged from 18 to 79, with an average of 48 years. Regarding the education level, 42% of participants have an upper secondary education degree, and 21% have a diploma in higher education. Finally, in terms of household income level, the majority of participants (51%) indicated a monthly income level between 1500 and €3499.

### 4.2. Measurement Model

Prior to testing our hypotheses, the validity of the measurement instruments used in our questionnaire was examined.

Table 4 shows PLS-SEM estimates for the reflective constructs (i.e., perceived quality and purchase intention). The values of Cronbach’s alpha and composite reliability (CR) were above the threshold of 0.70 [88], thus supporting the reliability of the measures. All factor loadings were above the recommended value of 0.708 [88] and significant at the 1% level. Similarly, the AVE values for perceived quality and purchase intention exceeded the minimum threshold of 0.5 [89]. Taken together, these results suggest that the convergent validity of both constructs is acceptable. Finally, the discriminant validity of both measurement scales was examined. The square root values of the average variance extracted are greater than the correlation between constructs, meeting the Fornell–Larcker criterion [89].

Regarding the formative constructs, results in Table 5 show that the weights of the indicators are all significant except for two indicators of moral legitimacy (“The PDO label is concerned with the environment” and “The PDO label is concerned with animal welfare”) and one indicator of cognitive legitimacy (“The PDO label is an official label ”). These indicators were not deleted since the corresponding correlation values were higher than 0.5 [88]. Additionally, the VIF (variance inflation factor) values were below the cut-off value of 5 [90], revealing the absence of a multicollinearity problem for all indicators.

### 4.3. Structural Model

As shown in Table 6, the values of R^2^ for perceived quality and purchase intention are 0.569 and 0.603, respectively, meaning that our model is able to predict 57% of the perceived quality and 60% of the purchase intention of the PDO cheese.

A detailed examination of the influence of the four dimensions of the PDO label’s perceived legitimacy reveals that not all of them have a significant effect on the perceived quality of the PDO cheese, as we had hypothesized.

Regarding the perceived quality of PDO cheese, results show that the PDO label’s pragmatic, regulative, and moral legitimacy positively influence the perceived quality, supporting H1a, H1b, and H1c, whereas the influence of cognitive legitimacy is not significant. Thus, H1d is not supported. More precisely, pragmatic and regulative legitimacy are the main predictors of the perceived quality (respectively, β **=** 0.345, *p* < 0.001, and β = 0.339, *p* < 0.001), followed by the moral legitimacy (β **=** 0.161, *p* < 0.01) which has a more modest impact on the perceived quality.

**Figure 2:**
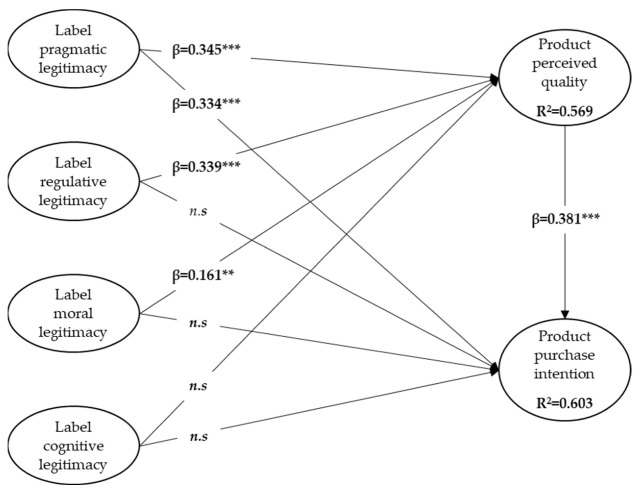
Results of the structural equation model predicting product perceived quality and purchase intention; *** *p* < 0.001, ** *p* < 0.01; *n.s.*: non-significant.

Similarly, pragmatic legitimacy, together with perceived quality, was found to directly influence consumers’ intention to purchase the PDO-labeled cheese positively (respectively, β = 0.334, *p* < 0.001, and β = 0.381, *p* < 0.001), supporting H2a and H3. Conversely, regulative, moral, and cognitive legitimacy did not have a direct significant influence on purchase intention. Therefore, H2b, H2c, and H2d are not confirmed. These results are summarized in Figure 2.

Looking at the indirect effects, results showed that both regulative and moral legitimacy (respectively, β = 0.129, *p* < 0.01 and β = 0.061, *p* < 0.01) indirectly influence the intention to purchase the PDO-labeled cheese through the perceived quality (Table 7). This means that the effects of regulative and moral legitimacy on purchase intention are fully mediated by perceived quality. Furthermore, perceived quality partially mediates the relationship between pragmatic legitimacy and purchase intention, given that pragmatic legitimacy has both a direct (β = 0.334, *p* < 0.001) and indirect (β = 0.131, *p* < 0.01) influence on purchase intention.

Overall, these results show that there is a difference in the impact of the different dimensions of the PDO label’s perceived legitimacy on the perceived quality of PDO cheese. Consumers build their perception of the label’s legitimacy by giving different relative importance to the dimensions of this legitimacy; priority seems to be given to the pragmatic dimension, followed by the regulative dimension, then the moral one. Consequently, as long as consumers perceive the pragmatic, regulative, and, to a lower extent, moral dimension of label legitimacy, the quality of the food product will be perceived as higher quality, which will, in turn, influence consumers’ purchase intention.

More precisely, our findings suggest that consumers, when considering whether or not to purchase a labeled food product, are similarly influenced by the perceived pragmatic legitimacy of the label and the perceived quality of the labeled product. These results and the lack of significant influence of the label’s cognitive legitimacy on the perceived quality and purchase intention will be discussed below.

## 5. Discussion

Our study on the perceived quality of PDO-labeled products highlights the influence of the pragmatic, regulative, and moral legitimacy of the PDO label on the perceived quality of PDO-labeled cheese. These results confirm the role of food quality labels on the perceived quality of food products and the ability of quality labels to reduce the perceived risk [1,12,13,14]. The label is perceived as a relevant quality signal, and its presence informs of a higher quality.

The perceived pragmatic legitimacy of the PDO label has a significant and positive influence on the perceived quality and purchase intention of the labeled product. Thus, the PDO label indeed acts as an informational shortcut for the consumer [11] insofar as the benefits that it provides are deemed positive and aligned with the collective interest. By providing reliable and readily accessible information, it aids in the evaluation of the quality and reinforces the intention to buy, presumably by creating a habitual buying behavior. Ultimately, it is a reference and a landmark that consumers search for when food shopping. Similar results are presented in a study by Śmiglak-Krajewska and Wojciechowska-Solis [91] on organic products, which point out that many consumers are not willing to perform any research while evaluating the organic offer and would rather look for a label that clearly conveys the key information about the quality of the products.

In the eyes of the French consumer, the fact that the label is used on food products seems to mean that it complies with standards, has proven its value, and is, therefore, trustworthy. Therefore, buying a product bearing the PDO label, which in addition is a public label, reduces the risk of a poor choice in terms of expected quality. Public labels have a tripartite guarantee system that ensures the independence of the prescriber, that is a public body, from the controller, and the producer. This result is consistent with previous research on trust in quality labels and their capacity to positively influence perceived quality.

Indeed, granting the PDO label is the result of a regulated and well-controlled process [34] that consumers seem to have understood insofar as the perceived regulative legitimacy has a significant influence on the perceived quality. This likely attests to a good understanding of what a PDO label is (particularly in the French context, where the history of quality labeling is long-standing) and of the requirements that apply to producers seeking to obtain the right to use it. Even though consumers are not able to identify the content of a PDO’s specifications, they have probably grasped the fact that these specifications can be restrictive for producers while also protecting consumers. Labels can be seen as signals of quality constructed based on a collective cognitive process of qualification that links producers and consumers and which are guaranteed by means of certification by a third party. In that sense, Karpik [92] considers labels as both judgment and promise devices. The regulative nature of the PDO label is, therefore, well recognized, certainly thanks to the intervention of independent third-party certifiers [32,93], which gives this type of label considerable credibility. The label has indeed a dual mission of guaranteeing compliance with some production and quality criteria to the consumer. However, the PDO label’s regulative legitimacy does not exert a significant direct influence on the intention to purchase the PDO-labeled cheese. The above is also supported by the results of the study conducted by Menozzi et al. [42], which shows that consumers’ trust in the PDO labeling system fails to predict their intention to purchase PDO cheese despite it being positively correlated with intention and behavior. One possible explanation is the existence of a cheese offer (besides PDO-labeled cheeses) that is considered to be of sufficient quality to satisfy consumers with a more constrained budget. The French food sanitary context is one of the most stringent in the world. In addition, it is also possible that other variables are affecting the purchase intention, such as the budget available for food and the budgetary trade-offs made by the consumer in a tense economic context, but this point was not examined in this research.

The perception of the PDO label’s moral legitimacy has a significant influence on the perceived quality of the PDO-labeled cheese but to a lesser extent in comparison with the two former dimensions. Thus, environmental issues (sustainable consumption, animal welfare, preservation of biodiversity) do appear as variables associated with the PDO label, but their influence is weaker on the perceived quality of the PDO-labeled cheese and indirect on the intention to buy. By its very nature, the PDO integrates certain aspects of more socially responsible production (animal welfare, preservation of local biodiversity, etc.), even if these aspects are not at the forefront of the discourse of the PDOs and producers (know-how and region-of-origin effect being the primary focus of communication), PDO label is a vector of good practices in both production and certification. However, the “moral” stance, i.e., the one that is imbued with a certain ideology (environment, animal cause, and fair remuneration of producers), seems to be secondary for PDOs. This may be explained by the advent of (non-PDO) labels dedicated to the environmental and/or animal cause and the fair remuneration of producers. Thus, consumers who are sensitive to these arguments would be more inclined to buy a product with these other labels that more explicitly express a moral commitment, as is the case for organic products, for instance [94]. Another explanation could be that French consumers are not yet fully aware of how their socially responsible purchasing behaviors could contribute to the societal challenges (agricultural systems) that we are collectively facing and, ultimately, to the installation of a new social order [47,54,58]. This resonates with previous research that specifically questions the shared social values [62,63,64,65] that can be embodied, inter alia, by a quality label.

**Figure 3:**
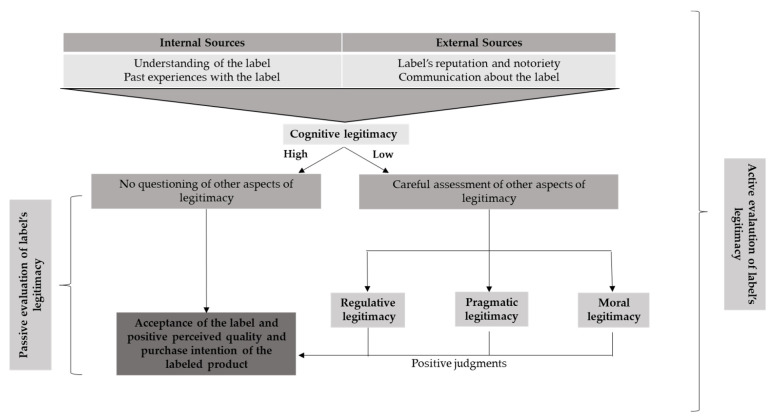
Process of assessing the legitimacy of quality labels by consumers. Source: authors.

Regarding the perceived cognitive legitimacy of the PDO label, our findings dy may, at first sight, seem puzzling since no significant influence on perceived quality and purchase intention was observed. One potential explanation is that the PDO label on cheese in France is “taken for granted“, cognitive legitimacy being defined here in line with the work on legitimacy by Suchman [49] and Alexiou and Wiggins [67]. Thus, the assessment of the PDO label’s cognitive legitimacy may not be conscious, and, therefore, the respondent will not be able to express it during the survey. Lastly, familiarity would not lead to attention but to the absence of attention; as much as the quality label would be “part of the landscape”, the consumer would not actively evaluate it. To draw a parallel, in a territory where the water is not safe to drink and where consumers have been drinking bottled water for many years, the perceived cognitive legitimacy of bottled water would probably be rooted in the unconscious, and consumers will not make the connection between the legitimacy of bottled water and the perceived quality or the intention to buy it since bottled water would be part of the landscape and would give rise to passive consumption behavior (cf. the work of Daniel Kahneman [95], Nobel Prize in Economics). This point needs to be confirmed by complementary studies carried out in other socio-economic contexts. Figure 3 summarizes our main results.

## 6. Conclusions

Our research broadens the scope of use of the concept of legitimacy in response to calls from researchers [96,97], who have highlighted the strategic interest of this concept borrowed from neo-institutional sociology in solving marketing problems.

This study contributes to the existing knowledge on consumer behavior toward quality labeled food products by testing the influence of the four dimensions of the legitimacy of the PDO label on the perceived quality and intention to purchase PDO cheese. By drawing on the literature on legitimacy, it provides an original approach to the perception of PDO labels and their influence on consumer behavior, which deserves to give rise to further research in order to document the link between perceived legitimacy, perceived quality, and purchase intention in other national settings and for other products.

On a practical level, our research provides insights into the lines of communication to be implemented by French producers and authorities. For example, producers could enhance the discourse of the PDO label around sustainable issues (environment, fair remuneration, animal welfare) by building on its moral legitimacy and by showing how the consumer, in choosing to buy a PDO-labeled product, can contribute to establishing a “new order” that is beneficial to all. More precisely, according to Calvo-Porral et al. [98], they could base their communication strategies on celebrity endorsement through the celebrities’ congruence with the PDO-labeled product, their credibility, and their trustworthiness, which has proven to be an effective tool in the food marketing field. Furthermore, applying blockchain to PDOs could also be a way of meeting the need for transparency (moral legitimacy) in the commitments made, which is already being explored for other certification schemes [99].

In conclusion, in a context where trust in information is increasingly questioned, trust in informational signals, such as the PDO label, deserves particular attention by making sure that the perceived reliability and legitimacy of official quality labels, as key landmarks for consumers, are maintained. This is the key role of trusted third parties, such as certification bodies. By highlighting their practices and processes more assertively, they would likely contribute to strengthening the normative (moral) legitimacy perceived by consumers but also by other stakeholders, including producers.

Limitations and further research: The main limitation of this study concerns the geographical scope and the fact that it is focused on a single product category, PDO cheese. As a matter of fact, the widespread use of the PDO label in France means that consumers are relatively familiar with it, which we assume has an impact on their perception and responses to the questionnaire. Nevertheless, these limitations call for the replication of this kind of study in other economic and socio-cultural contexts and on other food product categories (animal and plant-based) with quality labels. Another potential limitation of our study lies in the fact that certain variables (e.g., perceived risk, involvement, perceived expertise) that may moderate the influence of quality labels on consumers’ perceptions of labeled products were not taken into account so as not to complicate our research model or lengthen the questionnaire. Nonetheless, these moderating effects could nuance our results and, therefore, deserve to be explored in future work.

## Figures and Tables

**Table 1:** Dimensions of socio-political and cognitive legitimacy and their characteristics.

Dimension of Legitimacy		Definition
Socio-political legitimacy	Regulative legitimacy	- Is the result of compliance with the law or other forms of collective regulation; - Is based on the rules, norms, regulative processes created by influential actors, which give rise to consumers’ expectations.
	Pragmatic legitimacy	- Is based on an assessment by stakeholders of the value and benefits provided by the object evaluated; - Is judged based on results and actions; - Takes into account the social system and collective interest, (i.e., the actions undertaken are evaluated in alignment with the social system and interests, but with a calculated approach (almost self-interested) and not an axiological one).
	Moral or normative legitimacy	- Refers to ways of doing things (practices, structures, and processes), which are perceived as good and contributing to societal welfare (shared values).
Cognitive legitimacy		- Is based on cognition rather than interest or evaluation; - Is embedded in the collective subconscious and leads to being considered by stakeholders as taken-for-granted and inevitable; - Generates a perception of familiarity that leads to care (of the label for example).

Source: Adapted from Reynaux and Walas [69] and Bouhaddane [70].

**Table 2:** Constructs and indicators.

Construct	Indicators
Pragmatic Legitimacy	PL1. The PDO label helps me identify products with the characteristics I am looking for. PL2. The PDO label reinforces my choice. PL3. The PDO label provides me with a guarantee of compliance with production standards and requirements. PL4. The PDO label is a reference point for me.
Regulative Legitimacy	RL1. The PDO label is a symbol of rigorous quality control. RL2. The PDO label is controlled by accredited and independent bodies. RL3. The PDO label is based on a set of specifications drawn up by interested and competent parties. RL4. The PDO label is borne by stakeholders concerned with preserving the authenticity of their production. RL5. The PDO label complies with the conditions and requirements specified in the book of specifications.
Moral Legitimacy	ML1. The PDO label provides transparent communication to consumers about its purpose and objectives. ML2. The PDO label allows for a more sustainable consumption. ML3. The PDO label allows for a fairer remuneration of the producers. ML4. The PDO label is concerned with the environment. ML5. The PDO label is concerned with animal welfare. ML6. The PDO label contributes to the preservation of landscapes and biodiversity.
Cognitive Legitimacy	CL1. The PDO label has been consistent with its purpose since its inception. CL2. The PDO label is comprehensible. CL3. The PDO label is an official label. CL4. The PDO label has a good reputation among the general public. CL5. Using the PDO label to signal the quality of products to consumers is a given.
Perceived Quality	PQ1. PDO cheese is a high-quality product. PQ2. Compared to other cheeses without this label, a PDO cheese is a superior product. PQ3. The quality of a PDO cheese is better than that of other cheeses without this label.
Purchase Intention	PI1. If I had to buy cheese, I would probably buy a PDO cheese. PI2. If I needed cheese, I would certainly buy a PDO cheese. PI3. In the future, I will most likely buy a PDO cheese.

**Table 3:** Sociodemographic characteristics of the sample (n = 600).

Variables	Levels	%
Gender	Female	52.3
	Male	47.7
Age group (years)	18–24	7.7
	25–34	18.5
	35–44	17.2
	45–54	17.5
	55–64	21.7
	65 or older	17.5
Household size	1 member	20.5
	2 members	37.2
	3 members	17.0
	4 members	16.8
	5 or more members	8.5
Education level	Lower secondary/primary or below	5.7
	Upper secondary education	42.2
	Diploma of higher education	21.2
	Bachelor’s degree or equivalent level	15.8
	Master, postgraduate, or doctoral degree	14.9
	Prefer not to answer	0.2
Socio-professional category	Upper SPC	30
	Lower SPC	31
	Non-working	39
Household monthly net income	Less than €900	6.3
	€900–€1499	15.8
	€1500–€2499	26.8
	€2500–€3499	24.5
	€3500–€4499	16.5
	€4500 or more	10.0

**Table 4:** Reflective constructs: reliability and validity measures (factor loadings, Cronbach’s alpha (α), composite reliability (CR), and average variance extracted (AVE)).

Construct	Indicators	Loadings	α	CR	AVE	Correlation and Sqrt AVE ^1^	
						PQ	PI
Perceived Quality	PQ1	0.887 ***	0.856	0.861	0.664	0.815	-
	PQ2	0.749 ***					
	PQ3	0.803 ***					
Purchase Intention	PI1	0.882 ***	0.906	0.906	0.762	0.712	0.873
	PI2	0.883 ***					
	PI3	0.854 ***					

^1^ Square root of AVE (diagonal elements) and inter-construct correlation. *** *p* < 0.001.

**Table 5:** Formative constructs: validity measures (weights, correlations, and variance inflation factor (VIF)).

Construct	Indicators	Weights	*p*	Correlations	VIF
Pragmatic Legitimacy	PL1	0.203	0.009	0.861	2.691
	PL2	0.353	0.000	0.893	2.463
	PL3	0.237	0.003	0.798	1.904
	PL4	0.360	0.000	0.891	2.522
Regulative Legitimacy	RL1	0.295	0.000	0.842	2.045
	RL2	0.149	0.018	0.756	1.887
	RL3	0.326	0.000	0.882	2.425
	RL4	0.235	0.001	0.828	2.143
	RL5	0.191	0.023	0.826	2.322
Moral Legitimacy	ML1	0.424	0.000	0.871	1.838
	ML2	0.397	0.000	0.872	2.040
	ML3	0.165	0.020	0.711	1.720
	ML4	0.050	0.554	0.705	2.431
	ML5	0.039	0.634	0.644	2.027
	ML6	0.150	0.055	0.709	1.970
Cognitive Legitimacy	CL1	0.454	0.000	0.892	1.990
	CL2	0.234	0.003	0.754	1.646
	CL3	0.089	0.239	0.707	1.812
	CL4	0.202	0.018	0.798	2.109
	CL5	0.251	0.001	0.777	1.763

**Table 6:** Results of PLS-SEM estimation (R^2^ = coefficient of determination, β = unstandardized coefficients; S.E. = standard error; *p* = *p*-values).

Constructs	R^2^	β	S.E.	*p*
PQ predictors:	0.569			
PL		0.345	0.083	0.000
RL		0.339	0.081	0.000
ML		0.161	0.059	0.006
CL		−0.025	0.057	0.656
PI predictors:	0.603			
PL		0.334	0.090	0.000
RL		0.055	0.076	0.469
ML		0.033	0.058	0565
CL		0.058	0.069	0.395
PQ		0.381	0.075	0.000

PQ = Perceived Quality; PL = Pragmatic Legitimacy; RL = Regulative Legitimacy; ML = Moral Legitimacy; CL = Cognitive Legitimacy; PI = Purchase Intention.

**Table 7:** Indirect effects of the pragmatic, regulative, moral, and cognitive legitimacy on purchase intention.

Indirect Effects	β	S.E.	*p*
PL -> PQ -> PI	0.131	0.043	0.002
RL -> PQ -> PI	0.129	0.041	0.002
ML -> PQ -> PI	0.061	0.023	0.008
CL -> PQ -> PI	−0.01	0.022	0.658

PQ = Perceived Quality; PL = Pragmatic Legitimacy; RL = Regulative Legitimacy; ML = Moral Legitimacy; CL = Cognitive Legitimacy; PI = Purchase Intention.

## Data Availability

The data presented in this research will be available upon request from the corresponding author.

## Appendix A. Questionnaire

**Introduction**

Hello,

This survey is part of a strictly academic research project. It is intended to gather your opinions on food labels and your experience of consuming labeled cheeses. The questionnaire is anonymous and confidential. Please answer as frankly and spontaneously as possible. There are no right or wrong answers, only your personal views. Some questions may seem repetitive or abstract, but please answer them carefully.

The questionnaire will take about 10 min to complete. Thank you for your collaboration in this research, and we hope you will enjoy completing the questionnaire!

**I. TO GET TO KNOW YOU BETTER**

1. Gender
  - Male
  - Female
2. How old are you?
3. Please indicate in which municipality you currently live.
4. Please indicate your socio-professional category.
  - Agricultural operator
  - Craftsperson, tradesman and equivalent, business owner
  - Executive, higher intellectual profession
  - Intermediate profession (technician, foreman, technical officer, schoolteacher, nurse, instructor, etc.)
  - Employee (public or private sector)
  - Worker
  - Retiree
  - Student
  - Housewife or man/Other inactive
5. What is the highest level of education you have completed?
  - Lower secondary/primary or below
  - Upper secondary education
  - Diploma of Higher Education
  - Bachelor’s degree or equivalent level
  - Master, postgraduate, or doctoral degree
  - Prefer not to answer
6. Including yourself, how many people currently live in your household?
7. In which range does the net monthly income of your household fall?
  - Less than €900
  - €900–€1499
  - €1500–€2499
  - €2500–€3499
  - €3500–€4499
  - €4500 or more

**II. YOUR OPINION ON THE PDO LABEL**

8. What do you think of the PDO label?

(tick the box that best corresponds to your degree of agreement or disagreement with each of the statements below)

	**1. I Strongly Disagree**	**2. I Disagree**	**3. Neither Agree Nor Disagree**	**4. I Agree**	**5. I Strongly Agree**
The PDO label has been consistent with its purpose since its inception.					
The PDO label is comprehensible.					
The PDO label is an official label.					
The PDO label has a good reputation among the general public.					
Using the PDO label to signal the quality of products to consumers is a given.					
The PDO label helps me identify products with the characteristics I am looking for.					
The PDO label reinforces my choice.					
The PDO label provides me with a guarantee of compliance with production standards and requirements.					
The PDO label is a reference point for me.					
The PDO label provides transparent communication to consumers about its purpose and objectives.					
The PDO label allows for a more sustainable consumption.					
The PDO label allows for a fairer remuneration of the producers.					
The PDO label is concerned with the environment.					
The PDO label is concerned with animal welfare.					
The PDO label contributes to the preservation of landscapes and biodiversity.					
The PDO label is a symbol of rigorous quality control.					
The PDO label is controlled by accredited and independent bodies.					
The PDO label is based on a set of specifications drawn up by interested and competent parties.					
The PDO label is borne by stakeholders concerned with preserving the authenticity of their production.					
The PDO label complies with the conditions and requirements specified in the book of specifications.					

**III. YOUR EXPERIENCE OF PDO CHEESE CONSUMPTION**

9. Please indicate whether or not you agree with the following statements:

(tick the box that best corresponds to your degree of agreement or disagreement with each of the statements below)

	**1. I Strongly Disagree**	**2. I Disagree**	**3. Neither Agree Nor Disagree**	**4. I Agree**	**5. I Strongly Agree**
PDO cheese is a high-quality product.					
Compared to other cheeses without this label, a PDO cheese is a superior product.					
The quality of a PDO cheese is better than that of other cheeses without this label.					
If I had to buy cheese, I would probably buy a PDO cheese.					
If I needed cheese, I would certainly buy a PDO cheese.					
In the future, I will most likely buy a PDO cheese.					
